# Development and Evaluation of a Novel IoT Testbed for Enhancing Security with Machine Learning-Based Threat Detection

**DOI:** 10.3390/s25185870

**Published:** 2025-09-19

**Authors:** Waleed Farag, Xin-Wen Wu, Soundararajan Ezekiel, Drew Rado, Jaylee Lassinger

**Affiliations:** 1 Department of Mathematical and Computer Sciences, Indiana University of Pennsylvania, Indiana, PA 15705, USA; farag@iup.edu (W.F.); sezekiel@iup.edu (S.E.); kkpbc@iup.edu (D.R.); lhxdc@iup.edu (J.L.); 2 Department of Computer Science, University of Mary Washington, Fredericksburg, VA 22401, USA

**Keywords:** internet of things, machine learning, anomaly detection, port scanning, threat detection, testbed development

## Abstract

The Internet of Things (IoT) has revolutionized industries by enabling seamless data exchange between billions of connected devices. However, the rapid proliferation of IoT devices has introduced significant security challenges, as many of these devices lack robust protection against cyber threats such as data breaches and denial-of-service attacks. Addressing these vulnerabilities is critical to maintaining the integrity and trust of IoT ecosystems. Traditional cybersecurity solutions often fail in dynamic, heterogeneous IoT environments due to device diversity, limited computational resources, and inconsistent communication protocols, which hinder the deployment of uniform and scalable security mechanisms. Moreover, there is a notable lack of realistic, high-quality datasets for training and evaluating machine learning (ML) models for IoT security, limiting their effectiveness in detecting complex and evolving threats. This paper presents the development and implementation of a novel physical smart office/home testbed designed to evaluate ML algorithms for detecting and mitigating IoT security vulnerabilities. The testbed replicates a real-world office environment, integrating a variety of IoT devices, such as different types of sensors, cameras, smart plugs, and workstations, within a network generating authentic traffic patterns. By simulating diverse attack scenarios including unauthorized access and network intrusions, the testbed provides a controlled platform to train, test, and validate ML-based anomaly detection systems. Experimental results show that the XGBoost model achieved a balanced accuracy of up to 99.977% on testbed-generated data, comparable to 99.985% on the benchmark IoT-23 dataset. Notably, the SVM model achieved up to 96.71% accuracy using our testbed data, outperforming its results on IoT-23, which peaked at 94.572%. The findings demonstrate the testbed’s effectiveness in enabling realistic security evaluations and ability to generate real-world datasets, highlighting its potential as a valuable tool for advancing IoT security research. This work contributes to the development of more resilient and adaptive security frameworks, offering valuable insights for safeguarding critical IoT infrastructures against evolving threats.

## 1. Introduction

The Internet of Things (IoT) is transforming numerous sectors, including healthcare and manufacturing by connecting billions of devices and facilitating continuous data exchange across networks [1,2,3]. While these advancements unlock tremendous potential for automation, operational efficiency, and new user experiences, they expose IoT networks to cyber vulnerabilities. IoT devices, often constrained by limited processing power and memory [4,5]. Energy conservation and energy harvesting are applied to self-sustainable devices and resource-constraint edge nodes. A decisive task scheduling for energy conservation was proposed to achieve energy efficiency in IoT applications [4]. Lack of robust security architectures of IT systems due to the power and computational capability constraints makes them susceptible to cyberattacks [6]. These attacks range from data breaches and privacy violations to severe network disruptions such as Denial-of-Service (DoS) attacks, posing significant threats to critical infrastructures and undermining user trust. The cybersecurity risks highly threaten various IoT applications [7], including maritime power systems [8] and smart agriculture systems which contain smart devices such as unmanned aerial vehicles (UAVs) and unmanned ground vehicles (UGVs) [9,10]. To build reliable applications, the security challenges must be effectively addressed. Traditional cybersecurity solutions, initially designed for more homogeneous and predictable networked systems, are often ineffective when applied to dynamic, diverse, and resource-constrained IoT environments. Factors like the heterogeneity of IoT devices, resources constraints, variety of communication protocols, and complexity of application domains make implementing standardized security solutions across the board challenging. The need for adaptable and intelligent security solutions has made Machine Learning (ML) at the forefront, as a promising tool in IoT cybersecurity. Several ML models have been applied to IoT security and proved to be effective [11,12,13]. Specifically, the following models have been applied to enhancing IoT anomaly detection: Regression, Decision Tree, Naïve Bayes [11], Deep Convolutional Neural Networks (DCNN) [14], Extreme Gradient Boosting (XGBoost) [14,15,16], and Support Vector Machines (SVM) [17]. These ML models offer enhanced capabilities for anomaly detection, threat mitigation, and adaptive responses to evolving threats, positioning them as a critical element in addressing the unique security challenges of IoT [11,14,17].

Despite these advances, a critical gap remains in the availability of realistic, high-quality datasets for training and evaluating ML-based anomaly detection systems. Existing datasets such as Edge-IIoTset [1] and IoT-23 [14] are limited by privacy concerns, lack of diversity in device types, and insufficient representation of real-world traffic patterns. These limitations hinder the generalizability and robustness of ML models in practical IoT environments.

To expand upon the past efforts and further develop a reliable ML-based mechanisms for IoT, this research discusses the development of a physical testbed designed to simulate real-world scenarios akin to the Edge-IIoTset methodology, where Edge-IIoTset is one of the very few existing datasets. This testbed replicates environments like smart offices and homes, integrating various connected sensors, microcontrollers, and simulated attack vectors. This architecture enables it to emulate many cyberattack scenarios, including those documented in Edge-IIoTset and other IoT security research. The layered design of the testbed supports centralized data collection and monitoring, enabling ML algorithms to detect anomalies, flag potentially compromised devices, and evaluate mitigation techniques in a controlled yet realistic setting. This physical testbed also provides a platform for benchmarking the performance of the developed ML-based intrusion detection systems (IDS) against real-world datasets, such as those used in the Edge-IIoTset project. By examining the IDS’s robustness, consistency, and generalizability across varied environments, the study aims to establish a comprehensive evaluation framework for ML-based IoT security solutions. Additionally, ethical and privacy considerations, essential in any cybersecurity research, are embedded in the testbed’s design. Mechanisms for data anonymization, access control, and transparent data handling practices have been integrated.

This research is pivotal to the cybersecurity community as it addresses the critical gap in IoT security by providing a physical testbed capable of generating realistic, high-quality network traffic data. Traditional datasets in this domain are often limited by privacy concerns, data scarcity, and the challenge of replicating diverse IoT environments. By simulating a real-world smart office/home environment, this testbed not only facilitates the development and evaluation of ML models for anomaly detection but also offers a standardized platform for benchmarking their effectiveness. It empowers researchers and practitioners to study complex attack scenarios, evaluate mitigation strategies, and refine adaptive security frameworks without compromising user privacy. The testbed’s ability to mimic real-world conditions enhances the generalizability and robustness of ML-based security solutions, paving the way for more resilient IoT infrastructures. This contribution advances the field of IoT cybersecurity and serves as a foundational resource for the research community to explore innovative methodologies and drive impactful technological progress.

The novelty of our framework lies in its ability to generate diverse, realistic traffic from a heterogeneous IoT environment, enabling rigorous validation of ML-based IDSs under controlled yet authentic conditions. Unlike prior work, our testbed supports both benign and attack traffic simulation, integrates privacy-preserving mechanisms, and allows comparative benchmarking against existing datasets.

To validate the effectiveness of the proposed framework, we applied XGBoost and SVM models to the testbed-generated data and compared their performance to results obtained using the IoT-23 dataset [14,17]. The XGBoost model achieved a balanced accuracy of up to 99.977% on our dataset, closely matching its performance on IoT-23 (99.985%). Notably, the SVM model achieved up to 96.71% accuracy using our data, outperforming its results on IoT-23, which peaked at 94.572%. These results demonstrate the reliability and realism of our testbed and its value in supporting high-performance anomaly detection.

In summary, this paper presents the following key contributions:

- Development of a novel physical IoT testbed that emulates smart office/home environments, integrating diverse IoT devices and sensors to generate authentic network traffic.
- Simulation of realistic attack scenarios, including unauthorized access and network intrusions, to support the training and evaluation of ML-based anomaly detection systems.
- Provision of a controlled, ethically sound platform for data collection, enabling investigations of complex threat vectors without compromising user privacy.
- Benchmarking of ML algorithms using both the testbed-generated dataset and existing datasets, demonstrating the robustness and generalizability of the proposed intrusion detection system.
- Integration of privacy-preserving mechanisms such as data anonymization and access control, ensuring responsible handling of sensitive information.

The remainder of this paper is organized as follows: Section 2 presents a review of related work. Section 3 details the materials and methods used in developing the testbed, including hardware and software components. Section 4 presents the experimental results and performance evaluation of ML models. Section 5 discusses the implications of the findings, limitations, and future directions. Finally, Section 6 concludes the paper by summarizing the contributions and outlining potential avenues for further research.

## 2. Related Work

The development of robust IoT security frameworks has been significantly hindered by the scarcity of realistic, high-quality datasets [1,14,17]. Real-world IoT data is often sensitive and personal, making it difficult to collect and share without violating privacy. Even seemingly benign data can reveal confidential user information when aggregated, leading to heightened concerns over data misuse and breaches. These privacy risks, combined with the lack of standardized protocols and the rapid evolution of IoT technologies, make it challenging to create comprehensive datasets that reflect the diversity and complexity of real-world IoT environments [1,7].

Several benchmark datasets have emerged to support ML-based intrusion detection research. Edge-IIoTset is a comprehensive cybersecurity dataset designed to support machine learning research in Industrial and Internet of Things (IIoT) environments [1]. It was developed using a simulated edge computing architecture that integrates various IIoT devices and services. The dataset includes both benign and malicious traffic, covering a wide range of attack types such as Denial-of-Service (DoS), Man-in-the-Middle (MITM), and spoofing. Edge-IIoTset is structured to facilitate centralized and federated learning, and it provides labeled data suitable for training and evaluating intrusion detection systems. However, its simulated nature and moderate device diversity limit its ability to fully represent the complexity of real-world IoT deployments. IoT-23 [14,17] provides labeled traffic data for malware and port scanning attacks but lacks diversity in device types and does not incorporate privacy-preserving mechanisms. ToN_IoT [18] addresses heterogeneity and includes broader attack coverage, such as DDoS and data exfiltration, but remains simulation-based and offers limited privacy controls.

These limitations underscore the need for a physical testbed capable of generating realistic traffic while preserving user privacy. Our proposed testbed fills this gap by emulating a smart office/home environment with diverse IoT devices and sensors. It supports both benign and attack traffic generation, including port scanning, DoS, unauthorized access scenarios, and other attacks [19]. The testbed enables rigorous ML evaluation and integrates privacy-preserving features such as data anonymization and access control, offering a controlled and ethical platform for experimentation.

Beyond data generation, the testbed facilitates the study of IoT security, privacy, and interoperability. Smart buildings often integrate devices from multiple vendors, posing challenges for unified standards and protocols [7]. Our testbed allows simulation of such environments, enabling assessment of security mechanisms under realistic conditions. It also supports distributed architectures, aligning with the decentralized nature of modern IoT systems [2,7], and provides insights into optimizing security and privacy in multi-device contexts.

Furthermore, the testbed supports experimentation with ML models tailored to IoT networks, enabling effective detection of distributed DoS attacks and multi-stage threats. The quality of training data is critical to the performance of these models, and our testbed’s realistic traffic significantly enhances their accuracy and relevance. This capability is essential for building resilient IoT ecosystems that protect user privacy and maintain public trust.

The structured comparison in Table 1 above highlights the novelty and significance of our framework. By bridging the gap between simulation and physical deployment, our testbed offers a realistic, reproducible, and ethically sound platform for evaluating ML-based intrusion detection systems. It contributes to the advancement of secure, interoperable, and privacy-aware IoT infrastructures.

## 3. Materials and Methods

**Figure 1:**
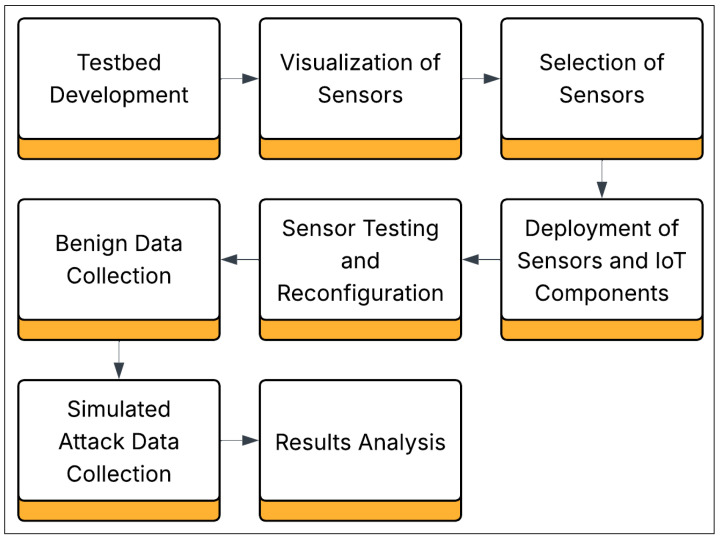
Block diagram showing the sequence of testbed development including selection and deployment of sensors, collections of benign and attack data, and analysis of results.

**Testbed Development:** To emulate a smart home or office environment, we developed detailed wiring schematics using constructed sensors from fundamental components through precision soldering, known as Fritzing, and implemented programming in the Arduino language. Each sensor was meticulously visualized in Fritzing during the design phase to ensure optimal circuit integrity. This hands-on approach to sensor construction allowed for a comprehensive understanding of the technical intricacies involved. Subsequently, the sensors were configured to collect and relay data to the central server autonomously. See Figure 1 below for a graphic depiction of the sequences of this project.

Our sensor network consisted of three distinct sensor types: an air quality sensor, a sound monitoring sensor, and an integrity sensor. These devices recorded data on environmental metrics, including air temperature, air quality, particle concentrations, and sound levels. Dispersed across several rooms or office spaces, the sensors continuously transmitted readings to the central server. Collected data were processed and displayed on a ThingsBoard dashboard, enabling streamlined analysis and real-time visualization accessible from remote locations. Additionally, the server monitored network traffic and archived all transmitted data including sensors’ data and network interactions, enabling us to seamlessly generate real-world IoT-based datasets. The sensor density was calibrated to reflect realistic traffic volumes observed in small-to-medium smart environments. While the current testbed uses a centralized server for data aggregation and monitoring, this design was selected to ensure controlled experimentation and ease of data collection. Future work will explore distributed architectures to assess scalability and resilience under decentralized conditions. This comprehensive dataset served as input for training our machine learning algorithm to identify anomalies, which could signal potential network threats or irregularities.

**Figure 2:**
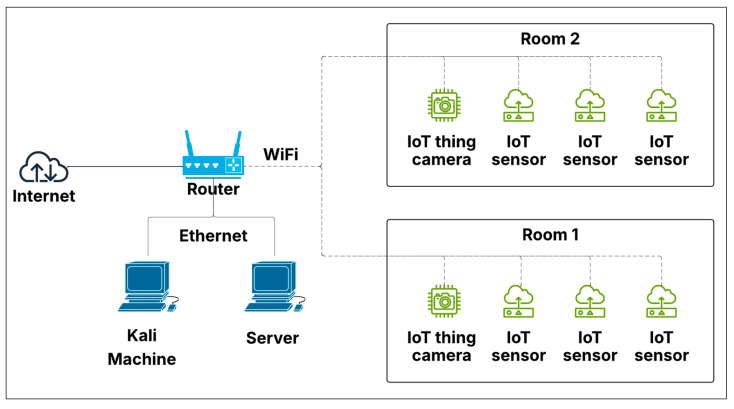
Initial IoT testbed topology spanning three rooms: Room 1 and Room 2 host IoT sensors and cameras, while Room 3 contains the central server and Kali Linux machine for attack simulation and data collection.

**Figure 3:**
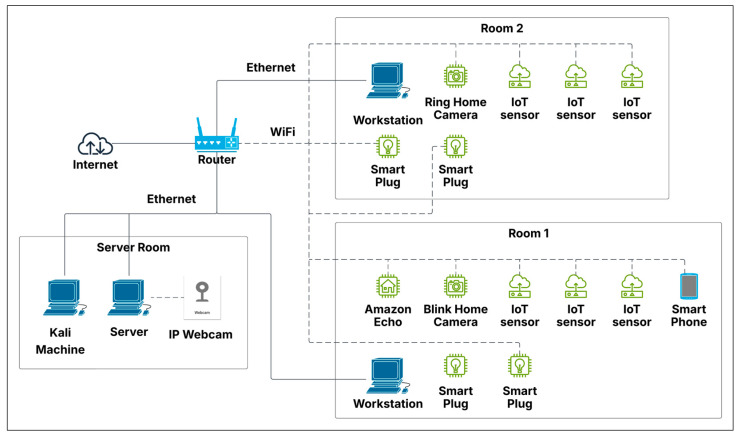
Updated testbed topology with three rooms. Room 1 and 2 contain IoT sensors, webcams, and workstations. Room 3 is the server room with a Kali Machine, server, and IP Webcam.

**Figure 4:**
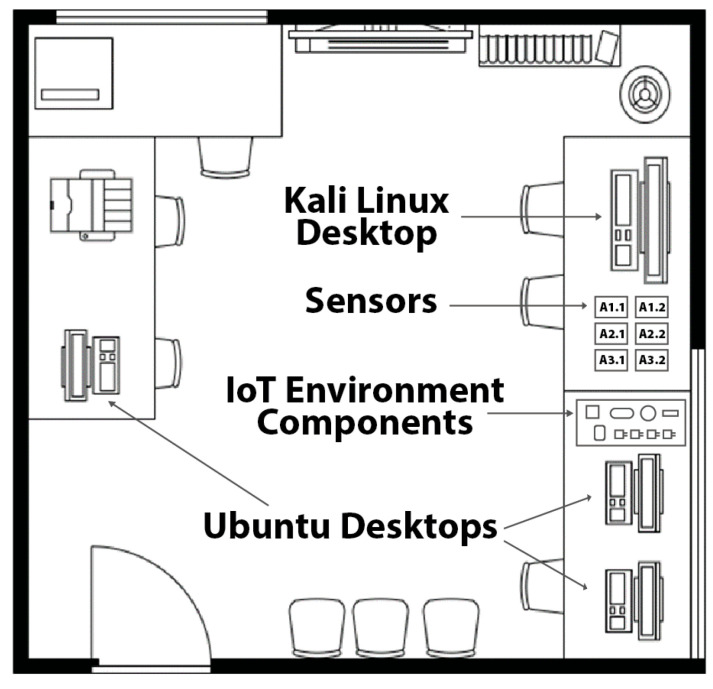
Setup of Room 1 which contains a Kali Linux desktop, sensors monitoring humidity, temperature, noise levels, and air quality, IoT Environment components including webcams and smart plugs, and three Ubuntu desktops.

Our initial testbed comprised a three-room configuration designed to replicate a compact office environment, as illustrated in Figure 2. The third room contained the central server, which managed sensor communications, alongside a Kali Linux (2024.1) machine designated as the compromised device for simulated attack scenarios. While preliminary tests yielded favorable results, the lack of network diversity led to anomalously high accuracy rates for all intrusion detection algorithms. Consequently, we restructured the entire setup to introduce greater complexity, further challenging the algorithms and connecting the network to the Internet. This updated testbed retained the three-room structure but now included additional devices in each room, such as smart wall plugs, security cameras, workstation computers, an Amazon Echo, and smartphones, as depicted in Figure 3. Figure 4 depicts the actual setup of one of the rooms that was used for this study.

**Figure 5:**
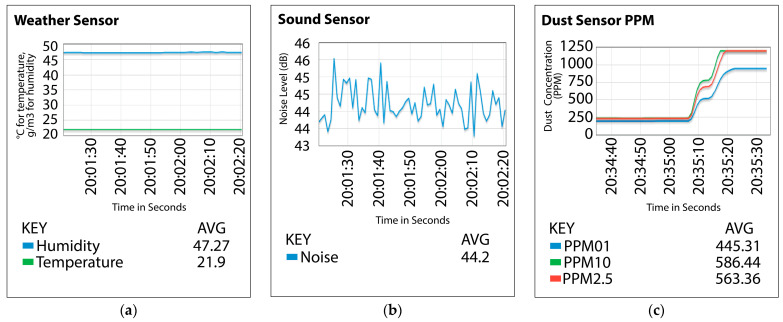
These charts depict (**a**) Humidity (g/m^3^) and temperature (°C) readings from sensors. (**b**) Noise (dB) level readings from sensors, and (**c**) Oxygen, CO_2_, and Particulate Matter (PPM) level readings from sensors.

Integrating these supplementary devices significantly enhanced our data collection by introducing novel features for algorithm evaluation. The selected devices, including smart plugs, webcams, environmental sensors, and voice-controlled assistants, are representative of those commonly deployed in smart homes and offices. Their integration ensures that the simulated environment mirrors realistic operational conditions, enhancing the relevance of the testbed for practical security evaluations. Sensor nodes connected seamlessly to the network, relaying data on environmental metrics like airborne particle concentrations, temperature, humidity, and sound levels. Data were processed and monitored in real-time via the ThingsBoard platform, providing live visualization and facilitating comprehensive asset monitoring as seen in Figure 5a–c, where x axes are time in seconds and y-axes are units of measured parameters (°C for temperature, g/m^3^ for humidity, and dB for sound level). Our methodical testbed construction yielded a realistic and scalable environment to evaluate IoT security across varied scenarios, advancing the development of more resilient and adaptive security protocols for IoT devices and networks.

**Software, hardware and sensors:** This section provides a comprehensive breakdown of the software and hardware components that form the technical foundation of the project. The documentation emphasizes the tools, platforms, and devices used, offering insights into their specific roles and contributions to the testbed’s functionality. The software discussion highlights key applications and operating systems, ranging from network analysis tools like Wireshark and Zeek to development platforms like the Arduino IDE. Each software component is integral to facilitating network traffic analysis, intrusion detection, and system management within the simulated environment. The hardware overview delves into the devices and sensors employed in constructing the testbed, including smart cameras, routers, and environmental sensors. These elements collectively replicate a realistic IoT ecosystem, enabling precise data collection, monitoring, and evaluation. The testbed includes devices from various manufacturers, such as TP-Link, Amazon, Logitech, and Wansview, each using different communication standards (e.g., Wi-Fi, Bluetooth, proprietary APIs). This multi-vendor setup enables the study of interoperability challenges and the evaluation of security mechanisms across heterogeneous systems. Special attention is given to the versatility and affordability of the selected components, ensuring the testbed remains accessible and reproducible for further research and application. By detailing these components, this section aims to provide a clear understanding of the technical infrastructure underpinning the project, laying the groundwork for the subsequent analysis and findings. Such details also provide guidance for other researchers interested in building and expanding our work.

**Software and hardware breakdown:** Emphasizing the primary tools and resources is essential for comprehensively understanding the project’s technical framework.

Below is a detailed description of each software component utilized in the project:

- ThingsBoard: An open-source IoT platform for data collection, processing, visualization, and device management.
- Wireshark: A network protocol analyzer that captures and decodes real-time network traffic for analysis.
- Zeek: A high-level network analysis framework designed to detect and analyze security issues.
- Brim: A user-friendly tool for visualizing and analyzing complex network data to identify threats.
- Hping3: A command-line tool for sending custom packets and testing network performance.
- Nmap: A security scanner used to discover network hosts, open ports, and running services.
- Arduino IDE: A development environment for programming and uploading code to Arduino microcontrollers.
- ISpy (Agent DVR): Open-source software for monitoring and recording activities with webcams or network cameras.

Below is a detailed description of each hardware component utilized in the project:

- TP-Link Archer A7 Router: Operates using OpenWRT for custom network configurations.
- Kali Linus Desktop: An OS for penetration testing and ethical hacking with specialized security tools.
- Ubuntu Desktop x3: A user-friendly open-source OS for diverse personal and professional applications.
- Logitech C920x HD Pro Webcam: A high-definition webcam for professional-grade video recording.
- Echo Dot 3rd Generation: A compact smart speaker powered by Amazon Alexa for voice-controlled operations.
- Kasa Smart Plug HS103P4 x4: Plugs for automating and monitoring power supply to devices.
- Wansview Wireless Security Camera: A smart camera with motion detection and night vision capabilities.
- Samsung s20 Ultra 5G: A smartphone providing robust connectivity and multimedia functions.
- Ring Home Camera: A security camera offering motion detection, night vision, and two-way audio.
- Arduinio IoT23: A versatile development board for building IoT applications with Wi-Fi and Bluetooth.
- Gravity: Laser PM2.5, O2 Sensor, and CO Sensor: Sensors for monitoring air quality and environmental conditions.
- DHT22 Temperature and Humidity Sensor: A device for measuring ambient temperature and humidity.
- Gravity Analog Sound Level Meter: A sensor for detecting and measuring ambient sound levels.

In summary, the project’s technical foundation integrates a diverse range of software and hardware components, each selected for their specific functionalities and relevance to IoT security. The software tools, including Kali Linux and Ubuntu (24.10), provide robust platforms for penetration testing, network analysis, and user-friendly operation. Meanwhile, the hardware components, such as webcams, smart speakers (Amazon Echo, Amazon, Seattle, WA, USA), sensors, and development boards enable the creation of a realistic and versatile IoT environment. Together, these elements establish a comprehensive framework that supports advanced research and practical applications in IoT security, fostering innovation and resilience in interconnected systems.

**Sensor Selection:** Creating substantial network traffic was essential for establishing a robust baseline of the network’s optimal performance and careful selection of sensors was pivotal to the success of our data collection efforts. Moreover, it was imperative to select practical sensors for our study—devices commonly integrated into smart office and home environments.

**Figure 6:**
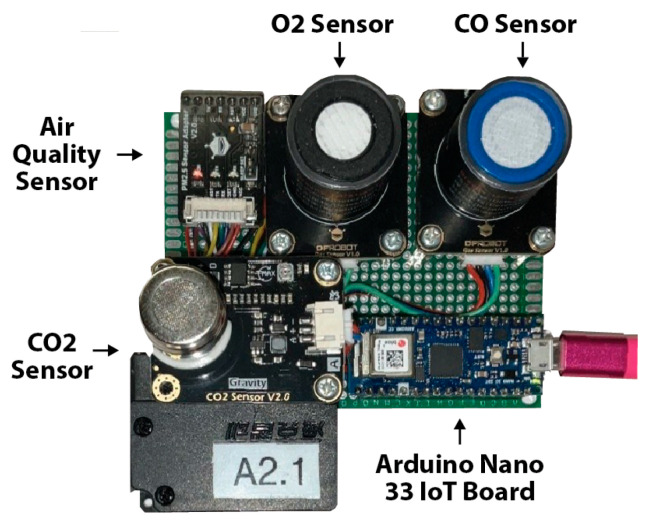
Photo of the A2.1 sensor which collects air quality, O2, CO, and CO_2_ levels using an Arduino 33 IoT Board. The A2.2 sensor (not pictured) is a duplication of the A2.1 sensor.

The sensors employed in our experiment, shown in Figure 6, are air quality sensors that measure the Parts Per Million (PPM) of Oxygen, Carbon Dioxide, Carbon Monoxide, and Particulate Matter (PM) 2.5 levels. Despite their compact size, low cost, and effectiveness in monitoring air quality, these sensors present limited security features. Their connection to the Internet renders them vulnerable to cyber adversaries who may exploit them for various types of attacks.

**Figure 7:**
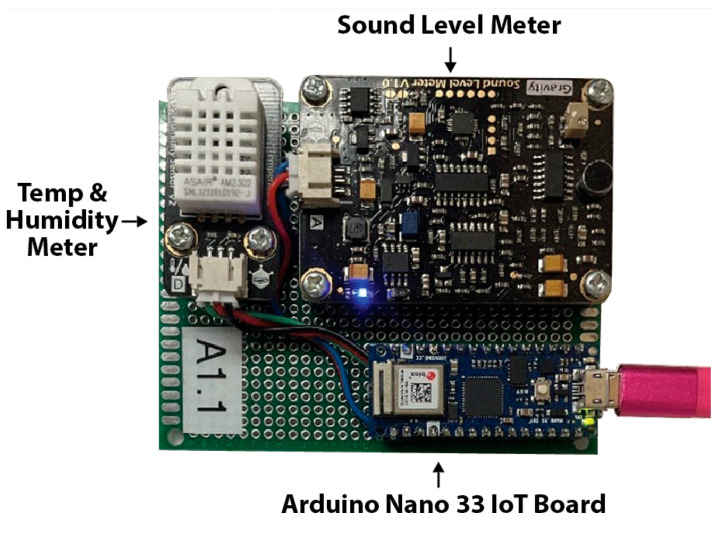
Photo of the A1.1 sensor which collects temperature, humidity, and sound levels using an Arduino 33 IoT Board. The A1.2 sensor (not pictured) is a duplicate of the A1.1 sensor.

Other sensors used in our study, shown in Figure 7, measure ambient temperature and humidity, with data capable of linking to central control systems to regulate heating, cooling, and humidity across different home or office areas. Additionally, Figure 7 shows a sensor module with sound monitoring capabilities that provides live decibel (dB) readings from different zones within the building. As with air quality sensors, these sensors are also vulnerable to exploitation by malicious actors.

**Figure 8:**
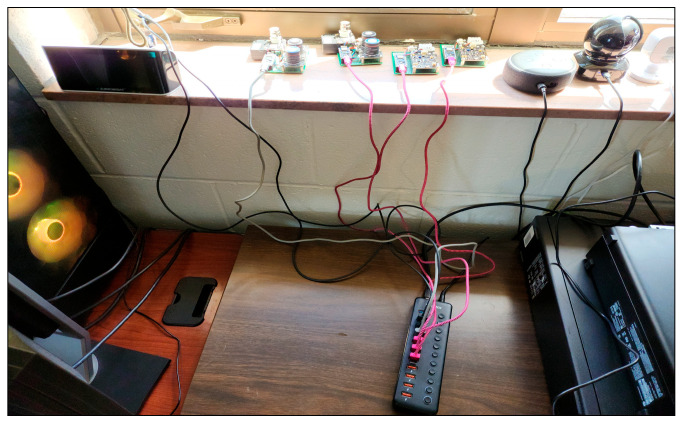
Photo taken in Room 1 showing sensors A1.1, A1.2, A2.1, and A2.2 collecting data on weather, sound, and air quality.

We ensured that our sensors incorporated standard functions in commercial IoT devices, such as data collection, transmission, and Internet connectivity. These functionalities include live video streaming, graphs, charts generated from the collected data and features similar to those of smart thermostats and doorbells, Figure 8 is a depiction of the physical sensors used in this study. Moreover, we prioritized keeping the cost of our sensor nodes low and their designs easy to replicate. This decision supports a broader scope of research possibilities for future studies, aligning with the commercial objectives of IoT devices and sensors to be affordable and widely accessible to encourage consumer adoption.

**Data Collection:** Data collection was structured into two primary categories: benign data collection and simulated attack scenarios. The first category aimed to capture network performance under optimal or near-optimal conditions, establishing a robust baseline for network behavior without interference. All sensors were integrated into the network to accomplish this, transmitting data continuously over several weeks while individuals carried out routine office activities. This extensive, uninterrupted data collection yielded a comprehensive baseline dataset of typical network activity, which is critical for training our ML anomaly detection models.

**Figure 9:**
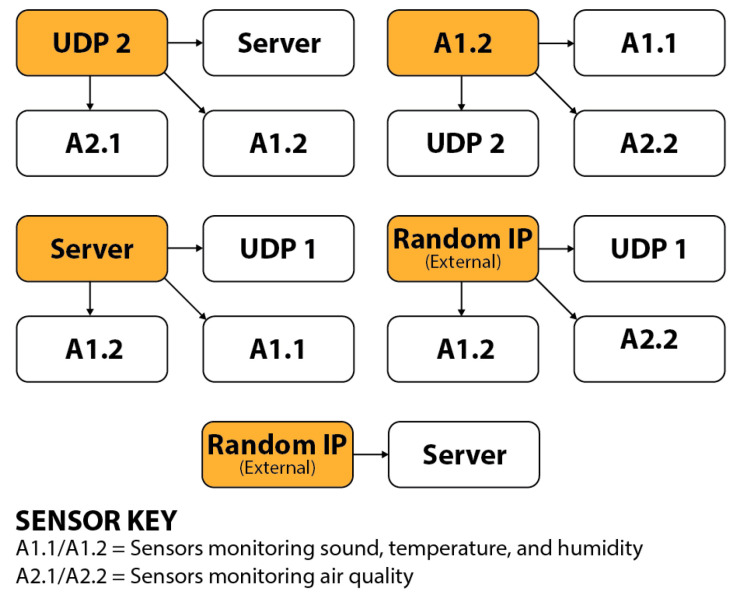
Detailed outline of attack parameters used to simulate diverse threat scenarios and collect data on benign and attack network traffic.

Once a strong baseline was established, we initiated a series of controlled network attacks to test the system’s anomaly detection capabilities. The initial attack simulated a vulnerability assessment through a port scan, where each network port was probed to identify those open to data transmissions, with the gathered information relayed back to the attacker. This port-scanning process was replicated across multiple sensors on the network, targeting specific devices and configurations to expose potential weaknesses. Following the port scans, a DoS attack targeting the identified unsecured devices was deployed. Various attack configurations simulated diverse threat scenarios, including attacks from compromised sensors targeting other network devices and servers and simulated external attacks on the network infrastructure. Figure 9 provides a detailed outline of all attack parameters. Using Wireshark on the server with port mirroring enabled on the router, we obtained a comprehensive view of all network activity and data transmissions, enabling precise monitoring and recording of benign and attack traffic.

## 4. Results

**Figure 10:**
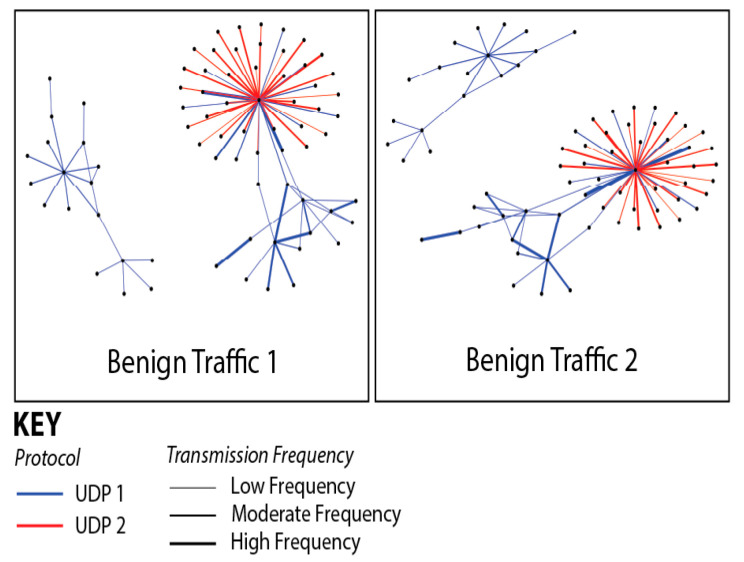
Composition of network tragic data. Colors indicate different protocols and line thickness represents transmission frequency between sensors.

**Figure 11:**
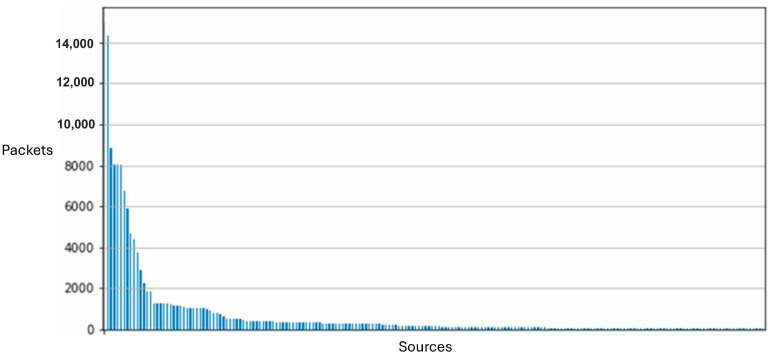
Network packet distribution across devices (i.e., packet sources) for the initial setup of the testbed. It shows that the vast majority packets were from a small number of sources.

**Figure 12:**
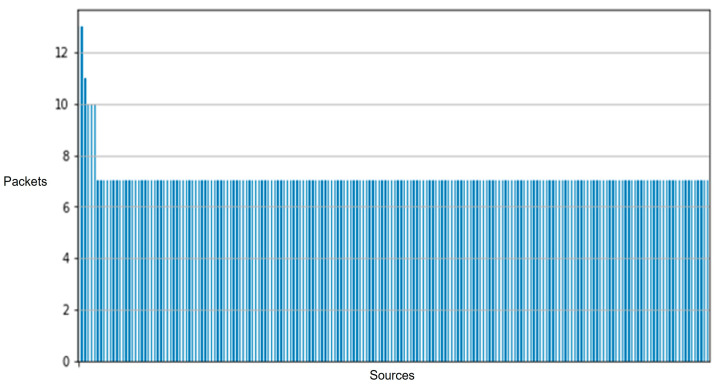
Network packet distribution across devices for the improved deployment of the testbed. It shows a more balanced distribution across diverse devices.

Figure 10 illustrates the composition of our network’s data traffic, with colors indicating the various protocols employed in data transmission and line thickness representing the frequency of transmissions between sensors. Our comparative analysis of two distinct data collection sessions revealed only minor outliers, affirming the consistency and stability of our dataset across multiple network captures. Figure 11 and Figure 12 demonstrate the impact of adding additional sensors to enhance network diversity. This adjustment significantly expanded the device count on the network, effectively reducing the influence of initial outliers and improving data robustness. Although our testbed accurately simulates a smart home or office environment, several variables, including environmental conditions and network load, could affect our system’s performance and data integrity. We conducted extensive tests across multiple data collection sessions and configurations within our testbed, each yielding distinct subsets of data. These variations allowed us to assess model consistency and robustness under different conditions, confirming consistent results and similar outcomes.

In addition, to verify the quality of data collected from our IoT testbed, we trained and tested the anomaly detection systems enabled by the ML models, Extreme Gradient Boosting (XGBoost) and Support Vector Machines (SVM), which are among the most accurate ML models for IoT anomaly detection, as proved in our previous works [14,17]. The results are summarized in the following table (Table 2).

To compare the performance results of anomaly detection using the data collected from our testbed with those using the IoT-23 dataset [14,17], we recall the results in our previous work [17]. Table 3 below presents the results of applying XGBoost and SVM with the IoT-23 dataset. For XGBoost, the balanced accuracy achieved across all data splits was 99.985%. As shown by Table 3, for SVM, the balanced accuracy reached 94.433% for an 80/20 split, 94.572% for a 75/25 split, and 94.521% for a 65/35 split. The split ratio indicates the portion of data allocated for training versus testing, with an 80/20 split signifying that 80% of the data were used for training and 20% for testing. Balanced accuracy, which is the average of sensitivity and specificity, reflects the model’s ability to detect positive cases (sensitivity) and negative cases (specificity). The high balanced accuracy across these results demonstrates robust model performance.

It is clear that (as shown in Table 2), when using the data collected from our testbed, for XGBoost, the balanced accuracy for an 80/20 split reached 99.977%; for a 75/25 split, the balanced accuracy reached 84.564% in the first run and 99.97% in the second. The 65/35 split achieved a balanced accuracy of 99.976%. The SVM’s balanced accuracy across various splits was 95.938%, 94.706%, 96.71%, and 96.467%, showing that the SVM model achieved a better performance when using the data collected from our testbed. These results affirm that both ML algorithms delivered strong performance across our generated dataset, showcasing the effectiveness of the developed testbed, and underscoring its reliability for anomaly detection in IoT environments.

In summary, our testbed was meticulously designed to replicate real-world conditions by accounting for network latency, device heterogeneity, and environmental variables that could influence system performance. By incorporating these elements into our testing process, we aimed to generate results that apply to and are representative of genuine smart home or office network environments.

## 5. Discussion

**Implications of the Physical Testbed for IoT Security Research:** The physical testbed developed in this study represents a significant contribution to the field of IoT cybersecurity. It overcomes several critical limitations found in prior work [1,18], namely, the scarcity of high-quality, real-world datasets and the ethical challenges of collecting such data in uncontrolled environments. By replicating a realistic smart home/office setting, the testbed ensures that generated network traffic closely mirrors actual IoT behavior while avoiding privacy violations. This positions the testbed as a reproducible, ethically sound platform that can support a broad range of experimental investigations in anomaly detection, intrusion response, and system resilience.

The modular nature of the testbed further increases its value. Researchers can incrementally add new devices, emulate distributed architectures, or simulate complex attack scenarios to study emerging threats. These capabilities enable fine-grained evaluations of ML-based intrusion detection systems (IDSs) under conditions that reflect real-world heterogeneity in IoT environments.

**Machine Learning Performance on Testbed-Generated Data:** The experimental results demonstrated that ML models, specifically XGBoost and SVM, achieved high balanced accuracy when trained on the testbed-generated dataset. Notably, these results were comparable to or slightly better than those obtained from the widely used IoT-23 dataset [14,17], validating the realism and diversity of the traffic captured in our environment. The performance metrics reflect the high quality of the collected data and its ability to reveal subtle anomalies that signal security breaches.

Interestingly, the initial configuration of the testbed yielded overly optimistic accuracy scores due to limited network diversity. This underscores the importance of realistic complexity in test environments; the subsequent inclusion of additional devices and Internet connectivity significantly improved the robustness and generalizability of model evaluation.

While our results demonstrate strong model performance, further exploration is needed to delineate the boundaries of resilience. Future experiments will involve stress-testing the models under varying network loads, environmental fluctuations, and adversarial noise to assess their robustness. Additionally, we aim to evaluate the fidelity of the simulated environment by comparing model behavior across different testbed configurations and attack types. These efforts will help quantify the generalizability and reliability of ML-based intrusion detection systems in diverse IoT contexts.

Although our current testbed focuses on known attack types, the anomaly detection models used, particularly XGBoost and SVM, are capable of identifying deviations from normal traffic patterns, which may include zero-day attacks. These models learn behavioral baselines and can flag previously unseen anomalies [20]. To strengthen this capability, future enhancements will incorporate unsupervised learning, ensemble approaches [15], and online learning techniques to improve detection of zero-day threats and support adaptive security responses.

The testbed’s architecture also allows for integration with existing IoT systems. Its compatibility with common communication protocols and device types enables it to ingest data from external networks, facilitating real-time training and deployment of anomaly detection models. This capability supports the practical application of the testbed in live environments, where it can be used to monitor traffic, identify threats, and initiate countermeasures dynamically. Future enhancements will focus on streamlining this integration to support continuous learning and adaptive security in operational IoT deployments.

Regarding computational complexity, the machine learning models used in our system, XGBoost and SVM, are not NP-hard in their standard forms. However, as the number of IoT devices and the volume of generated data increase, the training and inference processes may become more resource-intensive [14,17]. To address this, our testbed supports modular expansion and can be adapted to distributed architectures. Future work will explore edge-based processing and scalable learning techniques to ensure efficient operation in large-scale IoT environments.

While our testbed generated dataset reflects realistic traffic patterns, we acknowledge that its specificity, rooted in a particular smart office/home configuration, may pose challenges for broader generalization. Certain traffic behaviors and device interactions may be unique to our setup, potentially affecting model adaptability in other contexts. To address this, we introduced varied configurations and attack scenarios during data collection. Future work will focus on expanding the dataset across different environments and validating model performance in more diverse IoT deployments.

The expanded testbed configuration, with increased device diversity and Internet connectivity, was designed to emulate the complexity of real-world IoT deployments. In practice, IoT networks often consist of heterogeneous devices operating under varied protocols and usage patterns [7]. This diversity introduces non-uniform traffic flows and intermittent behaviors that challenge anomaly detection systems to distinguish between benign irregularities and genuine threats. Our testbed incorporated these elements to ensure that the ML models were exposed to realistic traffic variability. The strong performance of XGBoost and SVM under these conditions suggests that they are capable of adapting to heterogeneous environments, though further testing with even broader device types and attack vectors would be beneficial for assessing scalability and resilience.

The near-perfect accuracy achieved by XGBoost is likely a result of both the model’s inherent strengths and the quality of the testbed-generated dataset. XGBoost’s ensemble learning mechanism and ability to capture complex feature interactions make it particularly effective in structured anomaly detection tasks [15,16]. Additionally, the controlled nature of our testbed, while designed to replicate real-world conditions, ensures minimal noise and consistent traffic patterns, which may reduce variability and enhance model performance. Importantly, we mitigated the risk of overfitting by diversifying the network and introducing realistic attack scenarios, thereby validating the robustness of the results.

**Addressing Standardization and Interoperability:** IoT environments are inherently heterogeneous, often integrating devices with varying communication protocols and security features [7]. The developed testbed provides a flexible platform for testing how standardized protocols or lack thereof influence system security. It enables the study of interoperability challenges, particularly in multi-vendor smart building deployments, and facilitates experimentation with various configurations to evaluate their impact on anomaly detection performance.

Additionally, by supporting the simulation of distributed architectures, the testbed aligns well with contemporary IoT deployment trends [7], where edge computing and decentralized control are increasingly favored for scalability and privacy.

**Ethical and Practical Considerations:** A core strength of the proposed testbed is its attention to ethical and practical dimensions of cybersecurity research [1,14]. The controlled environment ensures that no real user Data are compromised, making the testbed suitable for iterative experimentation and educational use. Moreover, the focus on affordability and ease of replication supports broader adoption, including by institutions and researchers with limited resources.

The reproducibility of the testbed setup not only enhances transparency but also fosters collaboration and benchmarking across research groups, ultimately contributing to the development of more resilient and trustworthy IoT systems.

**Comparison with Related Literature:** Recent work on IoT energy efficiency and security provides a useful lens for situating our findings. For example, the authors introduced a task scheduling mechanism aimed at reducing energy consumption in IoT environments, highlighting the trade-off between resource optimization and computational overhead [4]. Our testbed complements this perspective by offering a platform where such trade-offs can be empirically assessed under realistic traffic conditions, thereby extending energy-optimization strategies into a broader security and anomaly detection context. Similarly, a framework presented in [5] addresses causes of energy inefficiency in IoT systems, emphasizing sustainability concerns. While their work focuses on system-level inefficiencies, our testbed-generated datasets enable researchers to evaluate how such inefficiencies manifest in network traffic patterns, potentially creating exploitable vulnerabilities. Thus, our results provide an orthogonal but complementary contribution, supporting both performance optimization and security enhancement.

From a security perspective, a comprehensive survey was published to discuss IoT threats and solution approaches [7]. Existing work underscored the scarcity of realistic, high-quality datasets as a major bottleneck in advancing effective intrusion detection systems [17,18]. Our testbed addresses this gap by generating diverse, reproducible traffic data under controlled conditions, thereby operationalizing many of the identified research needs. Similarly, the paper [8] surveyed cybersecurity in maritime power systems, while the recent publications [9,10] conducted systematic reviews on cybersecurity in smart agriculture. These domain-specific studies emphasize the unique vulnerabilities of critical infrastructures and cyber-physical systems. Our testbed extends this discourse by demonstrating how physical environments (e.g., smart offices or homes) can be realistically emulated to study heterogeneous device behavior and attack surfaces. Although our current focus is not sector-specific, the modular nature of the testbed allows it to be adapted to specialized domains such as agriculture, energy, or maritime contexts, bridging the gap between general purpose testbeds and sector-specific threat landscapes.

**Uncertainty in Input Data and Model Parameters:** As with any data-driven study, uncertainties arise from both the characteristics of input data and the configuration of machine learning models [12]. On the data side, environmental conditions (e.g., fluctuating noise levels, varying network loads, or device-specific idiosyncrasies) introduce variability into the collected traffic. While our multi-session data collection helped mitigate these effects by ensuring stability across runs, some residual uncertainty remains, particularly in scenarios with transient anomalies unrelated to malicious behavior. Another source of uncertainty stems from the heterogeneity of IoT devices; even within the same category, firmware updates or vendor-specific implementations may alter traffic patterns, limiting the generalizability of a fixed dataset.

Regarding machine learning model performance, hyperparameter tuning plays a crucial role in shaping detection accuracy and robustness [12,15]. Parameters such as learning rate, maximum depth (for XGBoost) [15], and kernel functions (for SVM) [17] were tuned using grid search; however, results remain sensitive to the search space and dataset splits. While we achieved consistently high balanced accuracy across multiple splits, minor variations underscore the need for systematic sensitivity analysis. Future extensions should employ techniques such as cross-validation, Bayesian optimization, or random search to quantify the robustness of models under different parameterizations. Additionally, the inclusion of advanced uncertainty quantification methods (e.g., Monte Carlo dropout or ensemble modeling) [15] would allow the system to assign confidence measures to predictions, providing more actionable insights in real-world deployments. Explicitly addressing these uncertainties will enhance the reliability and interpretability of ML-based intrusion detection in IoT environments.

**Limitations and Future Enhancements:** While the testbed successfully simulates many realistic attack scenarios, it has some limitations. The scale remains modest compared to large-scale IoT ecosystems in smart cities or industrial environments. Additionally, only a limited set of attack types, such as port scanning and DoS, were implemented. Future extensions should include more sophisticated adversarial tactics, such as stealthy multi-stage attacks, data poisoning, or lateral movement scenarios [7].

The modular architecture of our testbed allows for scalable expansion to simulate larger and more chaotic IoT networks. By adding additional rooms, devices, and network layers, the testbed can emulate the complexity found in smart cities or industrial IoT deployments. Furthermore, while our current attack scenarios focused on port scanning and DoS, the testbed is capable of supporting stealthier, multi-stage attacks such as lateral movement, privilege escalation, and data exfiltration. Incorporating these scenarios in future work will provide a more rigorous assessment of model resilience and detection accuracy under adversarial conditions.

Another area for improvement involves the integration of additional ML techniques such as deep neural networks [21], reinforcement learning [22], or federated learning [23] to assess their suitability in dynamic IoT contexts. Expanding support for these techniques may enhance adaptability and reduce false positives in real-time deployment scenarios.

## 6. Conclusions

This study demonstrates the potential of machine learning algorithms to significantly enhance IoT security through effective anomaly detection. By designing and implementing a physical testbed that emulates a smart office or home environment, we successfully gathered extensive network data, replicated realistic scenarios, and rigorously validated the performance of our developed detection algorithms. The integration of diverse IoT devices within the testbed highlighted the capability of machine learning models to detect a variety of attack vectors while maintaining operational efficiency. Employing a centralized server for data aggregation and processing further supported real-time monitoring and rapid threat identification, emphasizing the value of realistic environments for cybersecurity testing.

A central contribution of this research is addressing the critical scarcity of realistic IoT datasets by developing a physical testbed capable of generating high-quality, diverse network traffic data. This testbed replicates complex smart office and home scenarios, integrates multiple IoT devices and sensors, and simulates diverse cyberattack vectors, thereby providing an ethically grounded, reproducible, and scalable platform for training and evaluating ML-based intrusion detection systems. By filling this gap, our work empowers researchers and practitioners to benchmark security models against authentic conditions, advance anomaly detection capabilities, and contribute to building more resilient and adaptive IoT security frameworks.

While this research validates the application of machine learning in IoT security, it also reveals opportunities for further advancements. Future work could enhance the testbed by incorporating larger and more heterogeneous networks, multi-stage and stealth attack simulations, and varied network topologies to increase evaluation depth and realism. Refining the detection algorithms using advanced ML techniques such as deep learning and reinforcement learning could improve adaptability to evolving threats and reduce false positives. Additionally, exploring the transferability of these algorithms across different datasets and configurations would further demonstrate their robustness. Ethical considerations, including privacy preservation and data anonymization, must remain central, and integrating decentralized architectures could align the testbed more closely with real-world IoT ecosystems.

Overall, this research provides a practical and scalable framework to advance IoT security, offering insights that contribute to safeguarding critical infrastructures while supporting the broader fields of cybersecurity and data science. By pursuing these directions, we can help develop secure, trustworthy, and resilient IoT environments that protect users and maintain public trust in an increasingly connected world.

## Figures and Tables

**Table 1:** Comparison of IoT Security Testbeds and Datasets.

Framework/ Dataset	Architecture Type	Device Diversity	Attack Coverage	ML Evaluation Support	Privacy Considerations
Edge-IIoTset [1]	Simulated Edge/IIoT	Moderate	DoS, MITM, Spoofing	Yes	Limited
IoT-23 [14,17]	Simulated Network	Low	Port Scanning, Malware	Limited	None
ToN_IoT [18]	Simulated IoT/IIoT	High	DDoS, Data Exfiltration	Yes	Limited
Proposed Testbed	Physical Smart Office/Home	High	Port Scanning, DoS, Unauthorized Access	Yes	Integrated Anonymization and Access Control

**Table 2:** Balanced accuracy results with the dataset collected from our IoT testbed.

	80/20 Split	75/25 Split Run 1	75/25 Split Run 2	65/35 Split
XGBoost	99.977	85.564	99.970	99.976
	0.05 Sample	0.1 Sample	0.12 Sample	0.16 Sample
SVM	95.938	94.706	96.710	96.467

**Table 3:** Balanced accuracy results of IoT-23 dataset.

	80/20 Split	75/25 Split	65/35 Split
XGBoost	99.985	99.985	99.985
SVM	94.433	94.572	94.521

## Data Availability

Data are available upon request to the authors.

## Abbreviations

The following abbreviations are used in this manuscript:

IoT	Internet of Things
ML	Machine Learning
DoS	Denial-of-Service
IDS	Intrusion Detection System
PPM	Parts Per Million
O	Oxygen
CO_2_	Carbon Dioxide
PM	Particulate Matter
dB	Decibel
